# Efficacy and safety of nanosomal docetaxel lipid suspension-based chemotherapy in squamous cell carcinoma of the head and neck: A multicenter retrospective study

**DOI:** 10.3892/ol.2020.12207

**Published:** 2020-10-08

**Authors:** Saroj Kumar Das Majumdar, Sundaram Subramanian, Ghanashyam Biswas, Nisarg Joshi, Mujtaba A. Khan, Imran Ahmad

**Affiliations:** 1 Department of Radiotherapy, All India Institute of Medical Sciences, Bhubaneswar, Odisha 751019, India; 2 Department of Medical Oncology, VS Hospital, Madras Cancer Institute, Advanced Cancer Care, Chennai, Tamil Nadu 600031, India; 3 Department of Medical Oncology, Sparsh Hospital, Bhubaneswar, Odisha 751007, India; 4 Department of Medical Affairs and Clinical Development, Intas Pharmaceuticals Ltd., Ahmedabad, Gujarat 380054, India; 5 Jina Pharmaceuticals Inc., Libertyville, IL 60048, USA

**Keywords:** DoceAqualip, nanosomal docetaxel lipid suspension, squamous cell carcinoma of the head and neck

## Abstract

Squamous cell carcinoma of the head and neck (SCCHN) is the most common cancer in Indian men. Docetaxel alone or in combination with other chemotherapeutic agents is recommended for the management of SCCHN. The present multicenter, retrospective study was conducted to evaluate the efficacy and safety of a novel docetaxel formulation ‘nanosomal docetaxel lipid suspension (NDLS)’-based chemotherapy in SCCHN. The medical records of patients with SCCHN, who were treated with NDLS-based chemotherapy and followed up between August 2014 and September 2018, were reviewed. The efficacy endpoints were overall response rate [ORR; complete response (CR) + partial response (PR)] and disease control rate (DCR; CR + PR + stable disease) for patients receiving NDLS-based induction or palliative chemotherapy. Overall survival (OS) and safety were also evaluated. Efficacy evaluation was available in 30/34 patients (induction, 20/23; palliative, 10/11). NDLS-based induction chemotherapy showed an ORR and DCR of 95% and a median OS of 43.5 months (follow-up duration, 0.6–80.3 months). For NDLS-based palliative chemotherapy, the ORR and DCR were 50% and the median OS time was 4.6 months (follow-up duration, 1.8 to 14.3 months). At least one adverse event was reported in 82.6% patients. No new safety concerns were reported. Overall, NDLS-based chemotherapy was effective and well tolerated in the treatment of SCCHN.

## Introduction

Squamous cell carcinoma of the head and neck (SCCHN) develops in the mucous membranes of the mouth, nose and throat, and can include carcinomas of the oral cavity, floor of the mouth, tongue, tonsils, juxtatonsillar fossae, larynx and pharynx (1). In India, lip and oral cavity cancer is the second most common cancer (119,992 cases; 11.54%) after breast cancer; it is the most common cancer among Indian men (92,011 cases; 16.1%) and the fourth most common cancer in Indian women (27,981 cases; 4.8%) as per GLOBOCAN 2018 data (2). In total, 60–80% of SCCHN cases in India are diagnosed at advanced stages (3).

Early stage (I or II) SCCHN is usually treated with surgery and/or radiation therapy (RT), whereas multimodality treatment is generally required for patients with locally advanced (LA; stage III/IV) or metastatic SCCHN (4). The various treatment modalities include surgery followed by chemoradiotherapy (CRT), induction chemotherapy followed by CRT, RT or surgery (with or without adjuvant therapy), or epidermal growth factor receptor (EGFR) inhibition plus RT or CRT (5,6).

Docetaxel in combination with cisplatin and 5-fluorouracil (5-FU), the TPF regimen, is approved for the induction therapy of patients with LA SCCHN (7). The TPF and docetaxel/cisplatin (TP) regimens are recommended as induction/sequential chemotherapy for SCCHN (6). Docetaxel plus cisplatin/carboplatin, docetaxel plus cisplatin/carboplatin plus cetuximab and docetaxel monotherapy are recommended for the treatment of recurrent, unresectable or metastatic SCCHN (6).

Polysorbate 80 and ethanol vehicles in the conventional docetaxel formulation can cause acute hypersensitivity reactions, peripheral neuropathy, cumulative fluid retention, reactions at infusion sites, severe anaphylactoid reactions and alcohol intoxication (8–12). Nanosomal docetaxel lipid suspension (NDLS; DoceAqualip; Intas Pharmaceuticals Ltd.), a novel lipid-based formulation, which is free from polysorbate 80 and ethanol, was developed to overcome these toxicity issues (13).

NDLS is approved in India for the induction treatment of LA SCCHN (14). Other approved indications for NDLS include the treatment of patients with androgen-independent (hormone refractory) metastatic prostate cancer, advanced gastric adenocarcinoma, locally advanced or metastatic breast cancer after failure of prior chemotherapy and non-small cell lung cancer after failure of prior chemotherapy (14). NDLS was developed using lipids generally regarded as safe by the US Food and Drug Administration based on the patented ‘NanoAqualip’ technology [patent number: Worldwide (WO2008127358), Europe (2076244), Japan (5917789) and Canada (CA2666322)] (15). The NDLS development process includes the addition of docetaxel to high-pressure homogenized soy phosphatidylcholine and sodium cholesteryl sulfate in sodium citrate buffer under continuous high-pressure homogenization (13), resulting in nanosomal (<100 nm) particles of NDLS (13). The delivery of docetaxel to tumor tissues is increased with these nanosomal particles, helped by the already damaged tumor vasculature, which results in an enhanced permeability and retention effect. A greater systemic availability of docetaxel (13) is seen, hence leading to improved therapeutic outcomes in terms of response rates (16).

NDLS has shown efficacy and safety in the treatment of breast, ovarian, cervical, penile, gastric, hormone refractory prostate, non-small cell lung, head and neck cancers, and sarcoma (14,16–22). The present study reports a real-world, multicenter, retrospective account of the use of NDLS-based chemotherapy in the treatment of SCCHN.

## Materials and methods

### 

#### Study design, patient selection and endpoints

The present study retrospectively reviewed the medical records of patients with SCCHN who were treated with NDLS-based chemotherapy as part of their clinical care and followed up between August 2014 and September 2018 at multiple centers including All India Institute of Medical Sciences, Bhubaneswar (n=10), VS Hospital, Madras Cancer Institute, Advanced Cancer Care, Chennai (n=21), and Sparsh Hospital, Bhubaneswar (n=3), India. The study inclusion criteria were: i) Patients of all age groups and ii) both sexes, with iii) histopathologically or cytologically confirmed tumors, and iv) patients who received NDLS as part of routine clinical practice, who had at least one measurable lesion as per the Response Evaluation Criteria in Solid Tumors (RECIST) 1.1 (23). Patients who had cancer other than SCCHN were excluded from this report. The efficacy endpoints included: i) Overall response rate (ORR), defined as the total proportion of patients achieving complete response (CR) plus those with a partial response (PR); ii) disease control rate (DCR), defined as the proportion of patients achieving CR + PR + stable disease (SD); and iii) overall survival, defined as the time from treatment to death due to any cause. For patients who were still alive at the time of last follow-up (September 30, 2018) or who were lost to follow-up, OS was censored at the last recorded date that the patient was known to be alive. RECIST 1.1 was used for efficacy evaluation (23). Incidence of adverse events (AEs) were graded (where available) as per Common Terminology Criteria for Adverse Events 5.0 (24).

#### Ethics statement

The study was conducted after due approval from The OM Ethics Committee (Ahmedabad, India). The study was conducted in accordance with the ethical principles that have their origin in the Declaration of Helsinki (25), and in accordance with the International Conference on Harmonization's Good Clinical Practice guidelines (26), applicable regulatory requirements and in compliance with the submitted study protocol.

#### Statistical analysis

Demographic and baseline characteristics were summarized descriptively. Frequency and percentage were used for categorical variables and count, mean, standard deviation, median, minimum and maximum for continuous variables. The frequency and percentage of patients were used to present the response rates. Survival was analyzed using a non-parametric procedure performed using PROC LIFETEST (version 9.4; SAS Institute, Inc.). OS was measured using the Kaplan-Meier method and log-rank test. The AEs were summarized as frequencies and percentages by type of reaction.

## Results

### 

#### Patient disposition and demographics

In total, 228 patients with cancer who had received NDLS for their routine clinical care at different centers were evaluated. In the present report, the data of patients with SCCHN who received NDLS-based chemotherapy are presented.

Data of 34 patients with SCCHN, who were treated with NDLS-based chemotherapy regimens, were retrospectively analyzed. The baseline characteristics of these patients are summarized in Table I. The mean (SD) age of the patients was 54.70 (10.2) years and majority (73.52%) of the patients were men. Majority (61.76%) of the patients had stage IV cancer. All the patients had the Eastern Cooperative Oncology Group (ECOG) scores (27) of either 0 or 1. The most common anatomical sites for SCCHN were the mandible (n=9), buccal mucosa (n=7), hypopharynx (n=5), tongue (n=4), pharynx (n=2), oral cavity, oropharynx, parotid (n=1 for each) and not specified (n=4).

NDLS was administered as a 1-h infusion in 3-weekly cycles at 75 mg/m^2^ (n=29; 85.2%) and 50 mg/m^2^ (n=5; 14.8%); NDLS was used as first-line therapy in the majority (91.2%) of the patients. Granulocyte-colony stimulating factor was used in all the patients as primary prophylaxis.

As induction chemotherapy (n=23), 5 patients received an NDLS-based TPF (NDLS, platinum and 5-FU) regimen, 6 received NDLS plus cisplatin, 9 received an NDLS-based TPF plus nimotuzumab regimen and 3 received an NDLS-based TPF plus cetuximab regimen. An NDLS-based TP regimen (n=5), an NDLS-based TP plus nimotuzumab (n=3), an NDLS-based TP plus cetuximab regimen (n=2) and NDLS monotherapy (n=1) were used as palliative chemotherapy (n=11).

#### Efficacy

**Figure 1.**
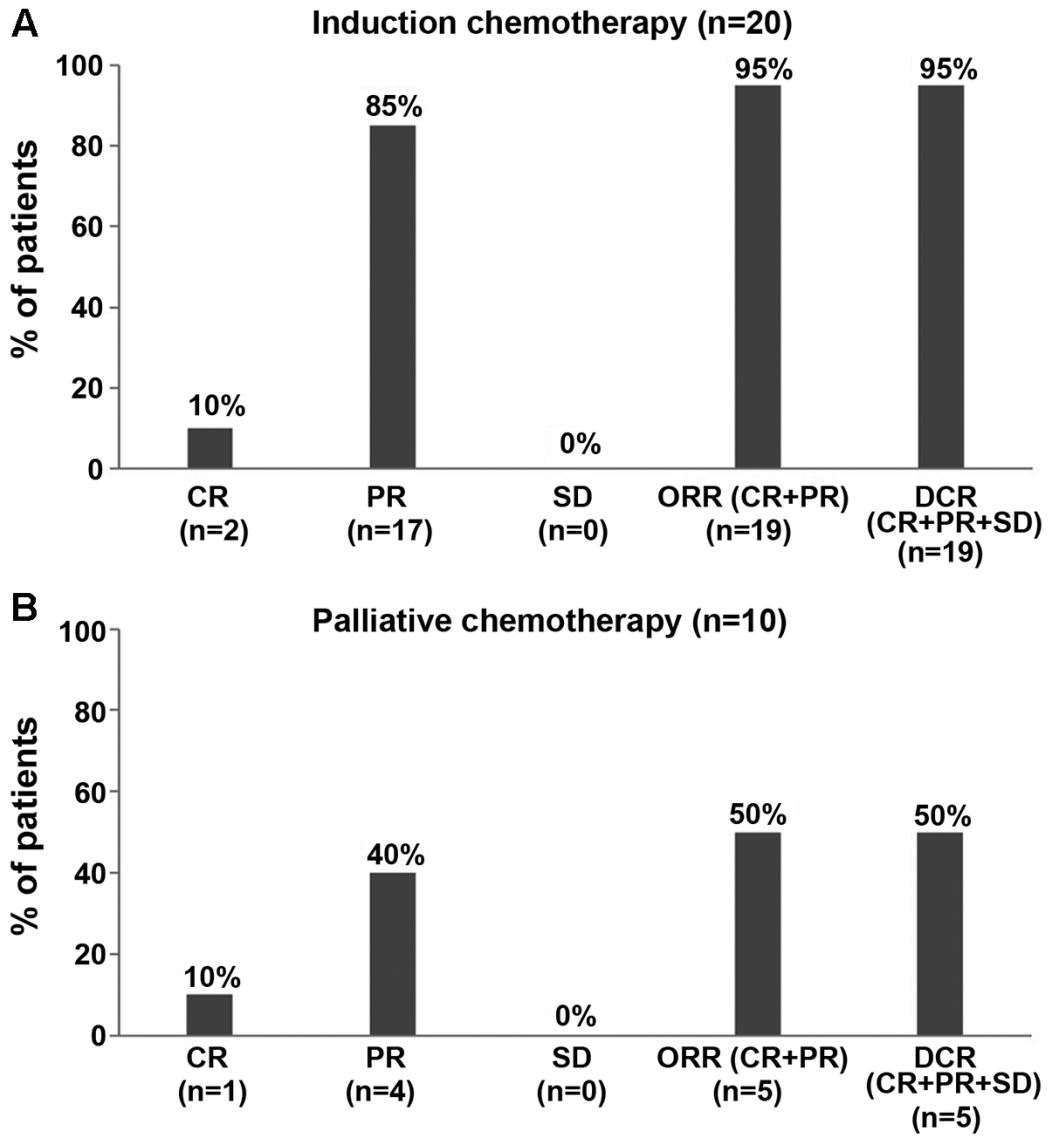
Efficacy of nanosomal docetaxel lipid suspension-based (A) induction (n=20) and (B) palliative (n=10) chemotherapy for the treatment of squamous cell carcinoma of head and neck. Disease progression was observed in 5 patients who underwent palliative chemotherapy and 1 patient who underwent induction chemotherapy. CR, complete response; DCR, disease control rate; NDLS, nanosomal docetaxel lipid suspension; ORR, overall response rate; PR, partial response; SD, stable disease.

Of the 34 patients who received NDLS-based chemotherapy for the treatment of SCCHN as induction and palliative chemotherapy, an efficacy evaluation was possible for 30 patients (induction, 20/23 patients; palliative, 10/11 patients). The ORR and DCR were 95% each for NDLS-based induction chemotherapy (CR=10%, n=2; PR=85%, n=17; Fig. 1A; Table II) and 50% each for NDLS-based palliative chemotherapy (CR=10%, n=1; PR=40%, n=4; Fig. 1B; Table II).

#### OS

**Figure 2.**
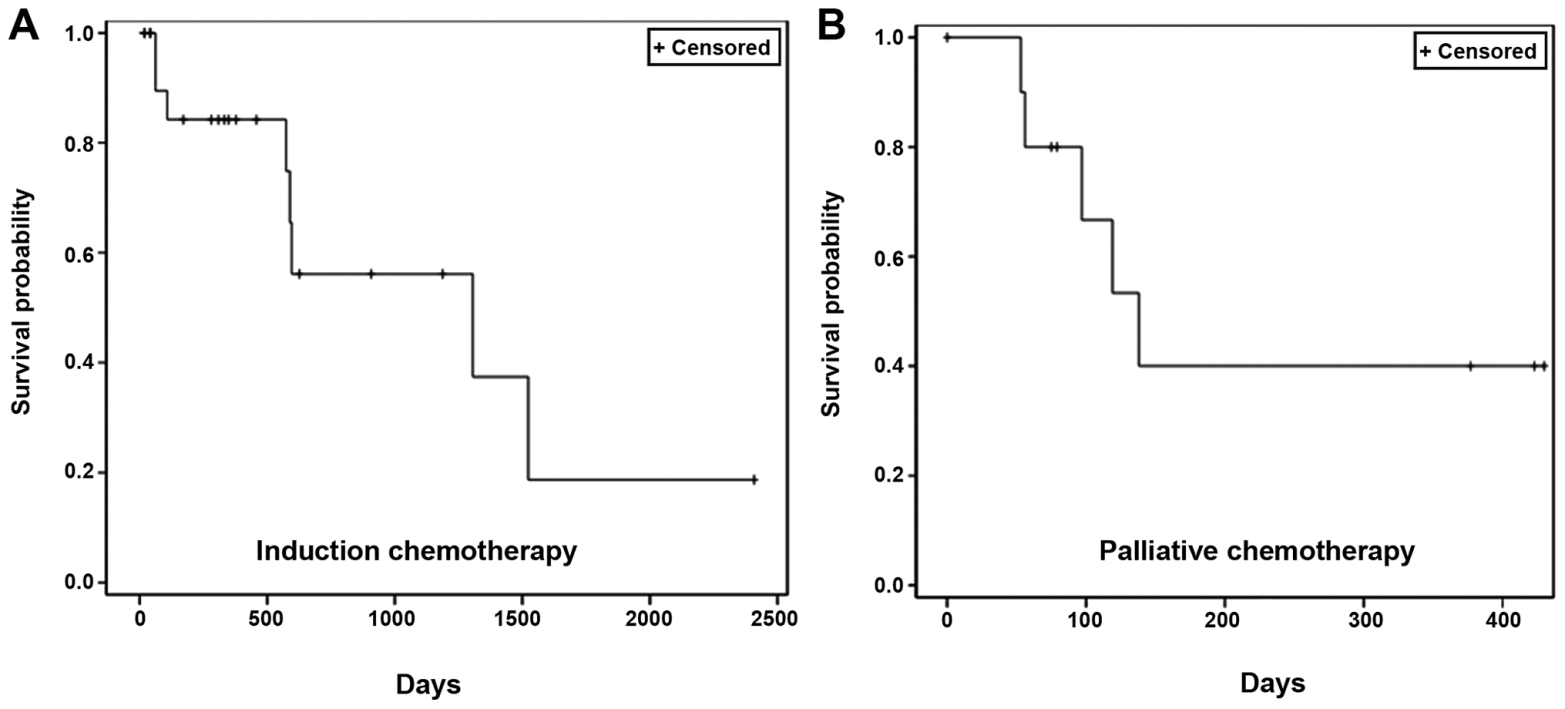
Kaplan Meier estimates of overall survival time in patients with squamous cell carcinoma of the head and neck treated with nanosomal docetaxel lipid suspension-based (A) induction (n=23) and (B) palliative (n=11) chemotherapy.

The patient survival data were collected from the administration of the first dose of NDLS-based therapy until the date of last follow-up (September 30, 2018) for patients who were alive and until the date of death for patients who died. The proportion of patients who were alive at the last follow-up was 65.2% (15/23 patients) in the induction setting and 54.5% (6/11 patients) in the palliative setting. In the induction setting, the median OS was 43.5 months (follow-up duration, 0.6–80.3 months; Fig. 2A). In the palliative setting, the median OS was 4.6 months (follow-up duration, 1.8–14.3 months; Fig. 2B).

#### Safety

The data on AEs was available for 23 patients, and at least one AE was reported in 19 (82.6%) of the patients. Grade 1 AEs were reported in 73.9% (17/23) patients, grade 2 in 13.0% (3/23) patients, grade 3 in 17.4% (4/23) patients and grade 4 in 4.3% (1/23) patients, respectively. Anemia, lymphopenia, thrombocytopenia and neutropenia were the reported hematological AEs, while hyperglycemia, constipation, nausea, vomiting and weakness were the most frequently reported non-hematological AEs (Table III). The grade 3/4 hematological AEs were neutropenia (8.7%), lymphopenia (8.7%) and thrombocytopenia (4.3%). One (4.34%) patient reported grade IV neutropenia.

## Discussion

The treatment modality for LA SCCHN includes induction chemotherapy or CRT with a cisplatin and 5-FU combination as the standard induction regimen (28). The addition of docetaxel to the standard TPF treatment as induction chemotherapy has shown significant survival benefits (1,29). The TPF regimen is recommended for the induction/sequential chemotherapy of SCCHN by the European Head and Neck Society (EHNC), European Society for Medical Oncology (ESMO), and European SocieTy for Radiotherapy and Oncology (ESTRO) guidelines (30,31). Furthermore, patients who achieve a CR or pathological CR after induction chemotherapy are likely to have a good prognosis (30).

An ORR of 68–87% has been reported with docetaxel-based induction chemotherapy for the treatment of SCCHN (32,33), whereas in the present study, NDLS-based induction chemotherapy demonstrated an ORR of 95%. The median OS with NDLS-based induction chemotherapy was 43.5 months (follow-up duration, 0.6–80.3 months). Docetaxel-based induction chemotherapy was previously evaluated in two phase III trials, TAX 323 (32) and TAX 324 (29). In the TAX 323 study, TPF induction chemotherapy (n=177) resulted in an ORR of 68% and a median OS of 18.8 months (32). Similarly, TPF induction chemotherapy in the TAX 324 study (n=255) resulted in an ORR of 72% and a median OS of 71 months (29). In a study by Pointreau *et al* (34), the TPF regimen (n=110) resulted in an ORR of 80%. In the present study, the NDLS-based TPF regimen was used in 5 patients and resulted in PR in 3 (75%, 3 out of 4 evaluated, NE=1 excluded) of these patients. Previously, neoadjuvant chemotherapy with docetaxel and cisplatin (n=34) demonstrated an ORR of 76.5% and a 3-year OS rate of 94.1% (35), compared with the 100% PR recorded in 6 patients (NDLS plus cisplatin) in the present study. Wang *et al* (33) used nimotuzumab, an anti-EGFR humanized monoclonal IgG1 antibody, in induction chemotherapy with the TPF regimen for LA SCCHN (n=31). This resulted in an ORR of 87.1% (30), compared with the 100% (CR, 2 patients; PR, 6 patients; 8 out of 8 evaluated, NE=1 excluded) recorded in the present study. In a phase III study, TPF induction chemotherapy followed by cetuximab showed a response rate of 78% (36), while the same regimen was used in 3 patients in the present study with 2 patients achieving a PR (100%, 2 out of 2 evaluated, NE=1 excluded).

Docetaxel is recommended as a first-line therapy for recurrent, unresectable or metastatic SCCHN as a single agent or in combination with cisplatin/carboplatin with/without 5-FU/cetuximab (6). In this setting, docetaxel-based chemotherapy has reported an ORR of 33–97% in the treatment of SCCHN (37,38). Patients receiving NDLS-based palliative chemotherapy demonstrated an ORR of 50% and a median OS of 4.6 months (follow-up duration, 1.8–14.3 months) in the present study. The Southwest Oncology Group evaluated the combination of docetaxel with carboplatin for advanced SCCHN (n=68) and reported a response of 25% and a median OS of 7.4 months (39), while the NDLS-based TP regimen (n=5) in the present study resulted in a CR and PR in 1 patient each.

In the conventional docetaxel formulation, polysorbate 80 and ethanol function as formulation vehicles, and have been implicated in AEs such as acute hypersensitivity reactions, cumulative fluid retention, peripheral neuropathy (8), severe non-immunological anaphylactoid reactions (9), reactions at injection sites (10) and alcohol intoxication (11,12). In the present study, neurotoxicity, fluid retention and acute hypersensitivity reactions were not reported with NDLS-based chemotherapy.

In the landmark TAX 323 study, neutropenia (76.9%), anemia (9.2%), thrombocytopenia (5.2%), febrile neutropenia (5.2%) and leucopenia (41.6%) were the grade 3/4 hematological AEs, whereas alopecia (11.6%), infections (6.9%), stomatitis (4.6%), lethargy (2.9%), diarrhea (2.9%), nausea, vomiting, neurotoxicity, anorexia and dysphagia (each 0.6%) were the grade 3/4 non-hematological AEs following the TPF regimen. In the TAX 324 study, neutropenia (55%), febrile neutropenia (4.8%), anemia (4.8%), thrombocytopenia (1.6%) and neutropenic infections (4.8%) were the grade 3/4 hematological AEs. Meanwhile, stomatitis (8.4%), nausea (5.6%), dysphagia (5.2%), anorexia (4.8%), vomiting (3.2%), diarrhea (2.8%), infection (2.4%) and lethargy (2%) were the grade 3/4 non-hematological AEs (29,32). TPF was the most common regimen used in the present study, and neutropenia (8.7%), lymphopenia (8.7%) and thrombocytopenia (4.3%) were the grade 3/4 hematological AEs observed. Grade 4 neutropenia was reported in 1 (4.34%) patient. Vomiting and weakness were the most frequently reported non-hematological AEs. Overall, NDLS was found to be well tolerated in patients with SCCHN.

Corticosteroids are routinely administered as a premedication to mitigate the toxicity issues of conventional docetaxel, such as hypersensitivity and the retention of fluid (40). In a recent study, Obradović *et al* (41) used transcriptional profiling of tumors and matched metastases in patient-derived xenograft mouse models and indicated the potential function of glucocorticoid receptor activation in the progression and metastasis of breast cancer. Corticosteroid premedication is not warranted with the NDLS formulation, especially when used as monotherapy, therefore avoidance of corticosteroids may help circumvent the risk of disease progression.

There are several limitations to the present study. Due to the retrospective design, the data for safety and survival are incomplete. The information pertaining to the history of tobacco use was not available in the medical records of all patients and hence could not be presented. Progression-free survival data could not be obtained, as these data and serial scans were not available for the majority of patients at most of the follow-up time points.

Overall, the NDLS-based therapy was effective and well tolerated in the management of SCCHN either as induction or palliative chemotherapy. The present data provides valuable insights into the effectiveness and safety of NDLS in the management of SCCHN. A clinical study is currently underway to validate the present findings.

## Figures and Tables

**Table I.** Disposition and baseline characteristics of patients treated with induction (n=23) and palliative (n=11) chemotherapy.

Parameters	All patients	Induction chemotherapy	Palliative chemotherapy
Mean age ± SD (range), years	54.70±10.2 (32–75)	55.91±10.3 (35–75)	53.61±9.26 (32–68)
Mean BSA ± SD, kg/m^2^	1.56±0.2	1.57±0.2	1.55±0.22
Sex, n (%)			
Male	25 (73.52)	16 (69.6)	9 (81.12)
Female	9 (26.47)	7 (30.4)	2 (18.18)
Cancer stage, n (%)			
I	2 (5.88)	2 (8.69)	–
II	5 (14.70)	5 (21.74)	–
III	6 (17.64)	6 (26.09)	–
IV	21 (61.76)	10 (43.48)	11 (100.0)
Metastasis site, n (%)^a^			
Lymph node	8 (23.53)	2 (8.69)	6 (54.54)
Lungs	1 (2.94)	–	1 (9.09)
Liver	1 (2.94)	–	1 (9.09)
Others	1 (2.94)	–	1 (9.09)
ECOG performance score, n (%)			
0	4 (11.76)	3 (13.04)	1 (9.09)
1	30 (88.23)	20 (86.96)	10 (90.91)
Comorbid disease, n (%)			
Hypertension	8 (23.52)	6 (26.09)	2 (18.18)
Diabetes	3 (8.82)	3 (13.04)	–
Others^b^	6 (17.64)	1 (4.35)	5 (45.45)

a Metastasis site available for 9 patients only.

b Other comorbid diseases included asthma, coronary artery disease, thrombophlebitis and dyslipidemia. BSA, body surface area; ECOG, Eastern Cooperative Oncology Group; SD, standard deviation.

**Table II.** Response rates based on NDLS based chemotherapy regimens.

Treatment regimen	No of patients treated	Response, n
Induction chemotherapy (n=23)		
NDLS-based TPF (NDLS, platinum and 5-FU)	5	PR=3, PD=1, NE=1
NDLS-based TP (NDLS plus cisplatin)	6	PR=6
NDLS-based TPF plus nimotuzumab	9	CR=2, PR=6, NE=1
NDLS-based TPF plus cetuximab	3	PR=2, NE=1
Palliative chemotherapy (n=11)		
NDLS-based TP (NDLS plus cisplatin)	5	CR=1, PR=1, PD=3
NDLS-based TP plus nimotuzumab	3	PR=2. PD=1
NDLS-based TP plus cetuximab regimen	2	PR=1, PD=1
NDLS monotherapy	1	NE=1

CR, complete response; NDLS, nanosomal docetaxel lipid suspension; NE, not evaluated; PR, partial response; PD, progressive disease; TP, taxane and platinum; TPF, taxane, platinum and 5-fluorouracil.

**Table III.** Safety profile of nanosomal docetaxel lipid suspension-based chemotherapy in squamous cell carcinoma of the head and neck (n=23).

AEs	All grades, n (%)
Hematological	
Anemia	14 (60.9)
Lymphopenia	7 (30.4)
Thrombocytopenia	6 (26.1)
Neutropenia	3 (13.04)
Non-hematological	
Hyperglycemia	5 (21.7)
Constipation	2 (8.7)
Nausea	2 (8.7)
Vomiting	2 (8.7)
Weakness	2 (8.7)
Anorexia	1 (4.3)
Diarrhea	1 (4.3)
Dyspnea	1 (4.3)
Hypotension	1 (4.3)
Mouth ulcer	1 (4.3)
Mucositis	1 (4.3)
Rash	1 (4.3)

AE, adverse event. AEs in different grades may occur in ≥1 patient; hence, the cumulative number of patients in different grades may exceed the total number of patients with individual AEs.

## Data Availability

The datasets used and/or analyzed during the current study are available from the corresponding author on reasonable request.
